# Pathological manifestation of the induced pluripotent stem cell‐derived cortical neurons from an early‐onset Alzheimer's disease patient carrying a presenilin‐1 mutation (S170F)

**DOI:** 10.1111/cpr.12798

**Published:** 2020-03-25

**Authors:** Ling Li, Hee Jin Kim, Jee Hoon Roh, Minchul Kim, Wonyoung Koh, Younghoon Kim, Hyohoon Heo, Jaehoon Chung, Mahito Nakanishi, Taeyoung Yoon, Chang Pyo Hong, Sang Won Seo, Duk L. Na, Jihwan Song

**Affiliations:** ^1^ Department of Biomedical Science CHA Stem Cell Institute CHA University Seongnam‐si Korea; ^2^ Neuroscience Center Samsung Medical Center Seoul Korea; ^3^ Department of Neurology Samsung Medical Center Sungkyunkwan University School of Medicine Seoul Korea; ^4^ Samsung Alzheimer Research Center Samsung Medical Center Seoul Korea; ^5^ Department of Neurology Asan Medical Center University of Ulsan College of Medicine Seoul Korea; ^6^ TOKIWA‐Bio, Inc. Tsukuba Center Inc. (TCI) Tsukuba Japan; ^7^ Dong‐A Socio R&D Center Dong‐A ST Yongin‐si Korea; ^8^ Theragen Etex Bio Institute Suwon‐si Korea; ^9^ Center for Clinical Epidemiology Samsung Medical Center Sungkyunkwan University School of Medicine Seoul Korea; ^10^ Department of Health Sciences and Technology SAIHST Sungkyunkwan University Seoul Korea; ^11^ Stem Cell & Regenerative Medicine Institute Samsung Medical Center Seoul Korea; ^12^ iPS Bio, Inc. Seongnam‐si Korea

**Keywords:** Alzheimer's disease, autophagy, induced pluripotent stem cells, mitochondrial dynamics

## Abstract

**Objectives:**

Alzheimer's disease (AD) is the most common neurodegenerative disease which is characterized by the formation of amyloid beta (Aβ) plaques and neurofibrillary tangles. These abnormal proteins induce disturbance in mitochondrial dynamics and defect in autophagy system. Since presenilin‐1 (PS1) is a core component in γ‐secretase complex, the mutations of PS1 gene cause the interference of γ‐secretase activity and lead to the increased Aβ_42_ secretion. We aimed to characterize the patient‐specific induced pluripotent stem cell (iPSC) line carrying PS1‐S170F mutation. Furthermore, we tested whether disease‐modifying drug can reduce AD pathology in the AD iPSC‐derived neurons.

**Materials and methods:**

Mononuclear cells (MNCs) were isolated freshly from the peripheral blood of an autosomal dominant AD (ADAD) patient carrying presenilin‐1 (PS1) mutation (Ser170Phe; PS1‐S170F) and a cognitively normal control. We generated induced pluripotent stem cell (iPSC) lines, which were differentiated into functional cortical neurons. Then, we measured the markers indicative of AD pathogenesis using immunocytochemistry and Western blot. We also investigated the mitochondrial dynamics in the AD iPSC‐derived neurons using Mito‐tracker.

**Results:**

We observed that both extracellular and intracellular Aβ levels were dramatically increased in the PS1‐S170F iPSC‐derived neurons, compared with the control iPSC‐derived neurons. Furthermore, PS1‐S170F iPSC‐derived neurons showed high expression levels of *p*‐Tau, which were detected both in the soma and neurites. The mitochondrial velocity in the PS1‐S170F iPSC‐derived neurons was much reduced, compared with that of the control. We also found a significant decrease of fusion‐related protein Mfn1 (membrane proteins mitofusin 1) and an increase of fission‐related protein DRP1 (dynamin‐related protein 1) in the PS1‐S170F iPSC‐derived neurons. We further observed the defects of autophagy‐related clearance in the PS1‐S170F iPSC‐derived neurons. Finally, we demonstrated the levels of Aβ and *p*‐Tau can be dramatically reduced by the treatment of LY‐2886721, a BACE1 inhibitor.

**Conclusions:**

Taken together, we have established and characterized the pathological features of an AD patient carrying PS1‐S170F mutation using iPSC technology, which will be the first case on this mutation and this iPSC line will serve as a useful resource for studying AD pathogenesis and drug screening in the future.

## 1 INTRODUCTION

Alzheimer's disease (AD) is the most common cause of dementia, which is pathologically defined by the accumulation of extracellular amyloid plaques and intraneuronal hyperphosphorylated tau aggregates associated with neuronal loss in the cerebral cortex.^1^, ^2^ Autosomal dominant AD (ADAD) is associated with mutations in presenilin‐1 (*PS1*), presenilin‐2 (*PS2*) or amyloid precursor protein (*APP*) genes. Amyloid beta (Aβ) peptides consist of 38‐43 amino acid residues and are generated from APP by beta (β)‐ and gamma (γ)‐secretase‐dependent sequential cleavages. PS1 gene acts as a core component in γ‐secretase complex, and the mutations in the PS1 gene interfere with γ‐secretase activity, resulting in the increase of Aβ42 secretion. The S170F in the PS1 gene is a well‐known mutation causing AD at very young age (ie third decade of life) with a rapid progression.^3^, ^4^, ^5^ We chose this mutation to generate iPSCs, because it is one of the most aggressive forms of AD both clinically and pathologically. Thus, we speculated that the iPSC‐derived neurons from this patient would exhibit AD pathology, including significant defects in mitochondrial and autophagy systems.

Induced pluripotent stem cell (iPSC) technology provides an excellent method for elucidating the molecular basis of human diseases.^6^, ^7^ An increasing number of studies have employed disease‐specific iPSCs in neurological diseases including AD,^8^, ^9^ and lots of them have investigated the phenotypes to model the AD in vitro*.* ^10^, ^11^, ^12^, ^13^, ^14^ Previous researches demonstrated that extracellular Aβ or phosphorylated tau‐induced impairment of synapses,^15^ disturbed mitochondrial dynamics^16^, ^17^, ^18^, ^19^ and defective autophagy system,^20^, ^21^, ^22^ and these compounding features of AD cannot be represented easily in other cellular model systems than iPSC system. Moreover, iPSC‐based disease modelling has the great potential towards personalized treatment as each iPSC line is patient‐specific and the response of drug treatments can be accurately monitored prior to human treatment.^7^, ^10^

In this study, we generated patient‐specific iPSCs from an AD patient carrying PS1‐S170F mutation for the first time using freshly isolated peripheral blood mononuclear cells (PBMCs). Then, we aimed to characterize the typical pathological features in the iPSC‐derived neurons from AD patients, compared with the normal control iPSC line which has been fully characterized in our previous study.^23^ We further investigated whether disease‐modifying candidate drug can reduce the pathological features of AD iPSC‐derived neuron.

## 2 MATERIALS AND METHODS

### 2.1 Participants

**FIGURE 1:**
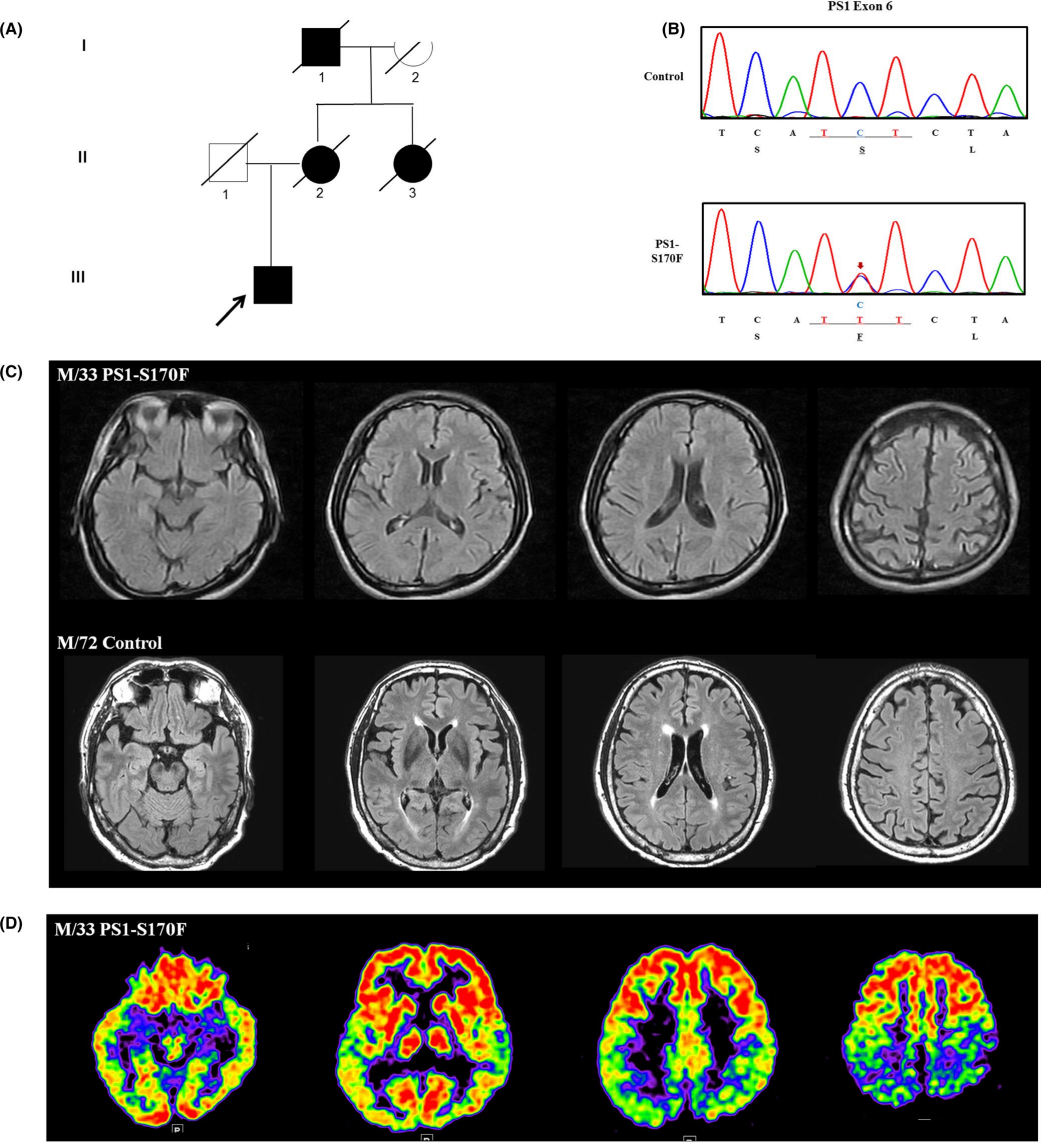
Clinical information of the patient with PS1‐S170F mutation. A, Pedigree showing autosomal dominant inheritance trait of early‐onset dementia. B, Genomic DNA sequences showing the presence of the heterozygous S170F mutation (CTC to TTT) in the PSEN1 gene only in the PS1‐S170F patient‐derived iPSC line. C, Fluid attenuated inversion recovery (FLAIR) MR images showing atrophy in the bilateral parietal areas. D, FDG‐PET showing hypometabolism in the bilateral temporo‐parietal areas

We collected blood samples from a 33‐year‐old Korean man who visited Samsung Medical Center in Seoul, Korea. He was clinically diagnosed as AD according to the criteria of National Institute on Aging‐Alzheimer's Association.^24^ The patient underwent neuropsychological test,^25^, ^26^ brain MRI and fluorodeoxyglucose (FDG)‐PET. As the patient had a strong family history of early‐onset dementia (Figure 1A), we screened for PS1 and performed apolipoprotein E (*APOE*) genotyping. As a parallel study, we also collected a blood sample from a healthy 72‐year‐old Korean man. He was proven to be cognitively normal on neuropsychological test.^25^, ^26^ He also underwent brain MRI, ^18^F‐florbetaben amyloid PET and APOE genotyping.

### 2.2 Characterization and differentiation of an iPSC line from the PS1‐S170F patient

Mononuclear cells (MNCs) were isolated freshly from the peripheral blood of the PS1‐S170F patient using the Ficoll‐PaqueTM PLUS method (GE Healthcare).^27^, ^28^ Isolated peripheral‐derived MNCs (PBMCs) were infected with SeVdp (KOSM) 302L.^27^, ^28^, ^29^ Genotyping of the PS1‐S170F single nucleotide mutation was performed by DNA sequencing (Cosmo Genetech). The PS1 gene was amplified by PCR (forward primer: ACA AAG TGA GAC CCT GTC T; reverse primer: CCA AGT ATG ACC TAT ATG TGG). Karyotyping, Sendai virus and mycoplasma detection PCR, teratoma formation assay, in vitro and cortical neuron differentiation were performed as we described before.^27^, ^28^

### 2.3 Reverse transcriptional polymerase chain reaction

We isolated total RNAs manually using the TRIzol reagent (Life Technologies) lysis and isopropyl alcohol precipitation. Complementary DNAs (cDNAs) were synthesized using a cDNA synthesis Kit (Cosmo Genetech), and reverse transcriptional polymerase chain reaction (RT‐PCR) amplification was performed in a final volume of 20 µL containing 200 ng/µL cDNA for each sample. Sequence information for the primers were as follows: GAPDH (forward primer: TGA CCA CAG TCC ATG CCA TCA CTG C; reverse primer: GTC ATA CCA GGA AAT GAG CTT GAC A); OCT4 (forward primer: CTG AAG CAG AAG AGG ATC AC; reverse primer: GAC CAC ATC CTT CTC GAG CC); NANOG (forward primer: TTC TTG ACT GGG ACC TTG TC; reverse primer: GCT TGC CTT GCT TTG AAG CA); SOX2 (forward primer: GCT GCA AAA GAG AAC ACC AA; reverse primer: CTT CCT GCA AAG CTC CTA CC); LIN28 (forward primer: CAC CAT GGG CTC CGT GTC CAA CCA GCA G; reverse primer: TCA ATT CTG TGC CTC CGG GAG CAG GGT AGG).^23^

### 2.4 Electrophysiological analysis

Whole‐cell patch recordings were performed as previously described,^30^ and we performed patch recording between 8 and 13 weeks after neural differentiation from the AD‐iPSCs. Briefly, before recordings, cells were transferred to a Nikon FN2 (Optical Apparatus) upright microscope fitted with a 40× water‐immersion objective, differential interference contrast (DIC), and infrared filter (IR) and perfused in aerated (95% O_2_/5% CO_2_) artificial CSF (ACSF) at room temperature (21‐25°C). The ACSF contained 124 mmol/L NaCl, 3 mmol/L KCl, 26 mmol/L NaHCO_3_, 1.3 mmol/L MgSO_4_, 1.25 mmol/L NaH_2_PO_4_, 2.4 mmol/L CaCl_2_ and 10 mmol/L glucose. Micropipettes (tip diameter, 1.5‐2.0 μm; 3‐6 MΩ) pulled from borosilicate tubing (P‐97; Sutter Instrument) and filled with an intracellular solution containing 143 mmol/L K‐glutamate, 10 mmol/L HEPES, 2 mmol/L KCL and 0.5 mmol/L EGTA, pH 7.2 ‐ 7.3 with KOH. Recordings were performed using EPC‐10 USB patch‐clamp amplifier (EPC‐10 USB; HEKA Electronik). Voltage control, current recording and filtration of current (at 1 kHz) were obtained using the EPC‐10 patch‐clamp amplifier (EPC‐10; HEKA Electronik) linked to a PC controlled and acquired with HEKA software. After the rupture of the cell membrane, leak conductance subtraction and series resistance compensation (70%‐80%) were performed and monitored periodically. In voltage‐clamp (VC) configuration, cells were given a series of voltage steps (duration, 50 ms) from −90 to +30 mV from a holding potential of −60 mV. In current clamp (CC) configuration, we measured the resting membrane potential, and then a negative holding direct current (in the range: −2 to −15 pA) was applied to bring the membrane potential to approximately −60 mV. This was done to compare neurons under identical conditions during AP firing. To assess the AP firing pattern in current‐clamp configuration, we applied a series of current steps (duration, 800 ms) from −20 to +60 pA. Current intensities were modified depending on the input resistance of each cell. Peak sodium current is defined as the maximal transient inward current at any command voltage. Peak potassium current was measured at 40 ms from the onset of the command voltage pulse. Summary data were expressed as mean ± standard error of the mean. Statistical analyses were performed using the 2‐tailed unpaired Student's *t* test and analysis of variance (ANOVA), respectively, with *P* < .05 considered significant.

### 2.5 Extracellular and intracellular amyloid‐β ELISA

We collected conditioned media (CM) from cultured neuronal cells (1 × 10^5^) at 48 hours after the last medium change from 4 to 10 weeks of differentiation for the measurement of extracellular Aβ levels. Intracellular Aβ levels were measured in a total of 1 µg proteins of TBS‐insoluble/SDS‐soluble fractions from 10‐week‐differentiated neurons. Aβ_40_ and Aβ_42_ levels were measured using the human Aβ_40_ and Aβ_42_ ELISA Kit according to the manufacture's instruction (IBL). ELISA plate reader (BioTek) was used to quantify Aβ_40_ and Aβ_42_ levels. All procedures were essentially same as described before.^27^, ^28^

### 2.6 Immunocytochemistry and Western blot

Immunocytochemistry and Western blot analysis were performed as we described before.^27^, ^28^ The following primary antibodies were used: anti‐OCT4 (1:200; Santa Cruz), anti‐SOX2 (1:200; Millipore), anti‐NANOG (1:200; R&D Systems), anti‐SSEA‐4 (1:100; Developmental Studies Hybridoma Bank), anti‐TRA‐1‐81 (1:100; Chemicon), Tuj1 anti‐tubulin beta III isoform (1:200; Millipore), anti‐SMA (1:100; DAKO); anti‐AFP (1:100; DAKO), anti‐Nestin (1:200; R&D Systems), anti‐Musashi (1:200; Millipore), anti‐Map2 (1:200; Millipore), anti‐TBR1 (1:100; Abcam), anti‐CTIP2 (1:100; Abcam), Aβ_42_ anti‐β‐Amyloid_42_ (1:500; Calbiochem), AT8 anti‐*p*‐Tau (1:1000; Thermo Fisher Scientific) and anti‐LC3B (1:500; Cell Signaling), Tau5 anti‐tau (1:1000; Thermo Fisher Scientific), anti‐Mfn1 (1:1000; Abcam), anti‐Mfn2 (1:1000; Cell Signaling), anti‐DRP1 (1:1000; Cell Signaling), anti‐Fis1 (1:1000; Santa Cruz), anti‐Ub (1:4000; Santa Cruz), anti‐LAMP2 (1:1000; Santa Cruz), anti‐Beclin1 (1:1000; Cell Signaling), p62 anti‐SQSTM1 (1:1000; Santa Cruz) and anti‐β‐actin (1:10 000; Santa Cruz).

### 2.7 Live cell imaging and mitochondrial dynamics analysis

We incubated the 10‐week‐differentiated neurons using the Mito‐tracker red (Thermo Fisher Scientific Cat.M7512) for 15 minutes before live cell imaging (LCI). Neurons were maintained at 37°C and were supplied with the atmosphere of 5% CO_2_/95% air (Live Cell Instrument) during imaging. Time‐lapse image recordings were acquired in 2‐second interval, and the duration was maintained up to 3.5 minutes. Quantitative analysis of mitochondria velocity was performed using KymographClear and KymographDirect.^27^, ^28^, ^31^ All procedures were essentially same as described before.^27^, ^28^

### 2.8 Drug treatment

LY‐2886721 (Selleck Chemicals) was prepared fresh from frozen stocks before administration and was dissolved in the differentiation medium, at 5 µmol/L as working concentration, as described previously.^27^, ^28^ Then, we treated the 6‐week‐differentiated iPSC‐derived neurons with LY‐2886721 for 5 days and investigated the effects on Aβ and *p*‐Tau levels.

### 2.9 Statistical analyses

All statistical analyses were performed using the one‐factor analysis of variance (ANOVA) following the Fisher's LSD (Least Significant Difference) in the Statistical Analysis System (Enterprise 4.1; SAS Korea). Significance was accepted at the 95% probability level. Data in graphs were presented as mean ± SEM. Statistical significance (ie* P* value) in the graphs were presented as follows: *P* < .05 (*), *P* < .01 (**) and *P* < .001 (***).

## 3 RESULTS

### 3.1 Case description

A 29‐year‐old man visited the memory clinic at Samsung Medical Center for 1 year of progressive memory impairment and visuospatial dysfunction. He was fired from his work due to serious memory problem and lost his way home from the workplace. He had 12 years of formal education and did not have any past history of medical problems. On neurological examination, he showed bradykinesia and tremor on the left side. On neuropsychological examination, he scored 25 on Mini‐mental state examination and showed a severe impairment on visuospatial function, verbal memory and visual memory tests (Table 1). He had a strong family history of early‐onset dementia on his mother's side (Figure 1). His mother was diagnosed to have dementia in her twenties and died at the age of 29 (Figure 1A, II‐1). His aunt was also demented in her thirties but did not visit the hospital (Figure 1A, II‐3). His grandfather was demented as he used to get lost in the neighbourhood in his forties (Figure 1A, I‐1). In genetic analysis, a known mutation in *PSEN1* (p.S170F, c.509C>T) was identified as heterozygous. The *APOE* genotype was ε3/ε3. The patient's brain MRI showed an atrophy in the bilateral parietal areas (Figure 1C), and FDG‐PET showed hypometabolism in the bilateral temporo‐parietal area, suggestive of AD (Figure 1D). He showed a rapid cognitive decline and scored 19 on MMSE 6 months after his first visit. He also developed gait disturbance and action induced myoclonus on the bilateral legs. Two years later, he entered nursing home as he became fully dependent on basic daily activities and died at the age of 34. We collected the patient's blood sample when he was 33 years old.

**TABLE 1:** Neuropsychological test result of the patient with PS1‐S170F mutation

	Raw score	*z*‐Score
Attention		
Digit span forward	5 (9)	−0.58
Digit span backward	3 (8)	−0.51
Language		
K‐BNT	49 (60)	0.21
Visuospatial		
RCFT copy	29 (36)	−3.87^a^
Memory		
SVLT immediate recall	9 (36)	−2.84^a^
SVLT delayed recall	0 (12)	−3.34^a^
SVLT recognition	18 (24)	−1.55^a^
RCFT immediate recall	10 (36)	−0.66
RCFT delayed recall	0.5 (36)	−2.74^a^
RCFT recognition	7 (24)	−5.97^a^
Frontal‐executive		
COWAT phonemic	37 (45)	2.54
Stroop colour reading	94 (112)	0.28
MMSE	25 (30)	−1.97^a^

Note

Abbreviations: COWAT, Controlled Oral Word Association Test; K‐BNT, Korean version of the Boston Naming Test; MMSE, mini‐mental state examination; RCFT, Rey‐Osterrieth Complex Figure Test; SVLT, Seoul Verbal Learning Test.

^a^ Cognitive domain that showed low performance expected for the patient's age and education.

The cognitively normal control showed no atrophy on brain MRI (Figure 1C), and his ^18^F‐florbetaben amyloid PET showed no significant amyloid deposition in the brain. His *APOE* genotype was e3/e4. This study was approved by the Institutional Review Board of Samsung Medical Center [1044308‐201612‐BR‐031‐05]. We obtained a written consent from each participant and his next of kin.

### 3.2 Generation of an iPSC line from the AD patient carrying PS1‐S170F mutation and differentiation into cortical neurons

Isolated MNCs were reprogrammed using Sendai virus vector (SeVdp) which expresses four reprogramming factors (OCT3/4, SOX2, cMYC and KLF4).^23^, ^29^ In our iPSC generation procedure, we routinely pick more than three individual clones and select the best growing clone among them for further analyses, including neuronal differentiation experiments. We generated and characterized a patient‐specific iPSC line from the AD patient carrying PS1 mutation (Ser170Phe; PS1‐S170F) for the first time (Figure S1A‐G). In this study, we compared the PS1‐S170F iPSC line with the normal control iPSC line which has been fully characterized in our previous study.^23^ PS1‐S170F patient‐derived iPSC line exhibited the typical expression of undifferentiated pluripotent stem cell markers, such as OCT4, SOX2, SSEA4 and TRA‐1‐81 (Figure S1A), which did not carry a SeV integration (Figure S1F), compared with the control iPSC line. Genotyping of the established iPSC line was confirmed using a conventional sequencing method (Figure 1B). Differentiation potential of iPSC lines was assessed in vitro by three‐germ layer marker expression (Figure S1B) and in vivo by teratoma formation (Figure S1C).

To characterize the cortical neurons derived from the iPSC lines, we established the cortical neural differentiation protocols by modifying previous procedures (Figure S2A).^12^, ^23^ Differentiated cells expressed general neural precursor cell (NPC) (ie Nestin, SOX2 and Musashi) and neuronal markers (ie Tuj1 and Map2), as well as cortical neuron markers (ie TBR1 and CTIP2) (Figure S2B,C). To examine the functional differences between the control and PS1‐S170F iPSC‐derived neurons, we used the whole‐cell patch clamp to record the differentiated neurons. All of the differentiated neurons exhibited spontaneous repetitive action potentials (AP) of currents clamp mode at 10‐12 weeks after differentiation (Figure S2D), indicating that all of the 10‐week‐differentiated neurons generated neuronal signal responses and were matured into functional neurons in vitro. However, there was no significant difference in the amplitude of AP (mV), sodium and potassium current (pA) between two iPSC lines (Figure S2E‐H), suggesting that there was no prominent difference in the differentiation propensity between the control and PS1‐S170F iPSC‐derived neurons.

### 3.3 Increased extracellular and intracellular Aβ levels in PS1‐S170F iPSC‐derived neurons

**FIGURE 2:**
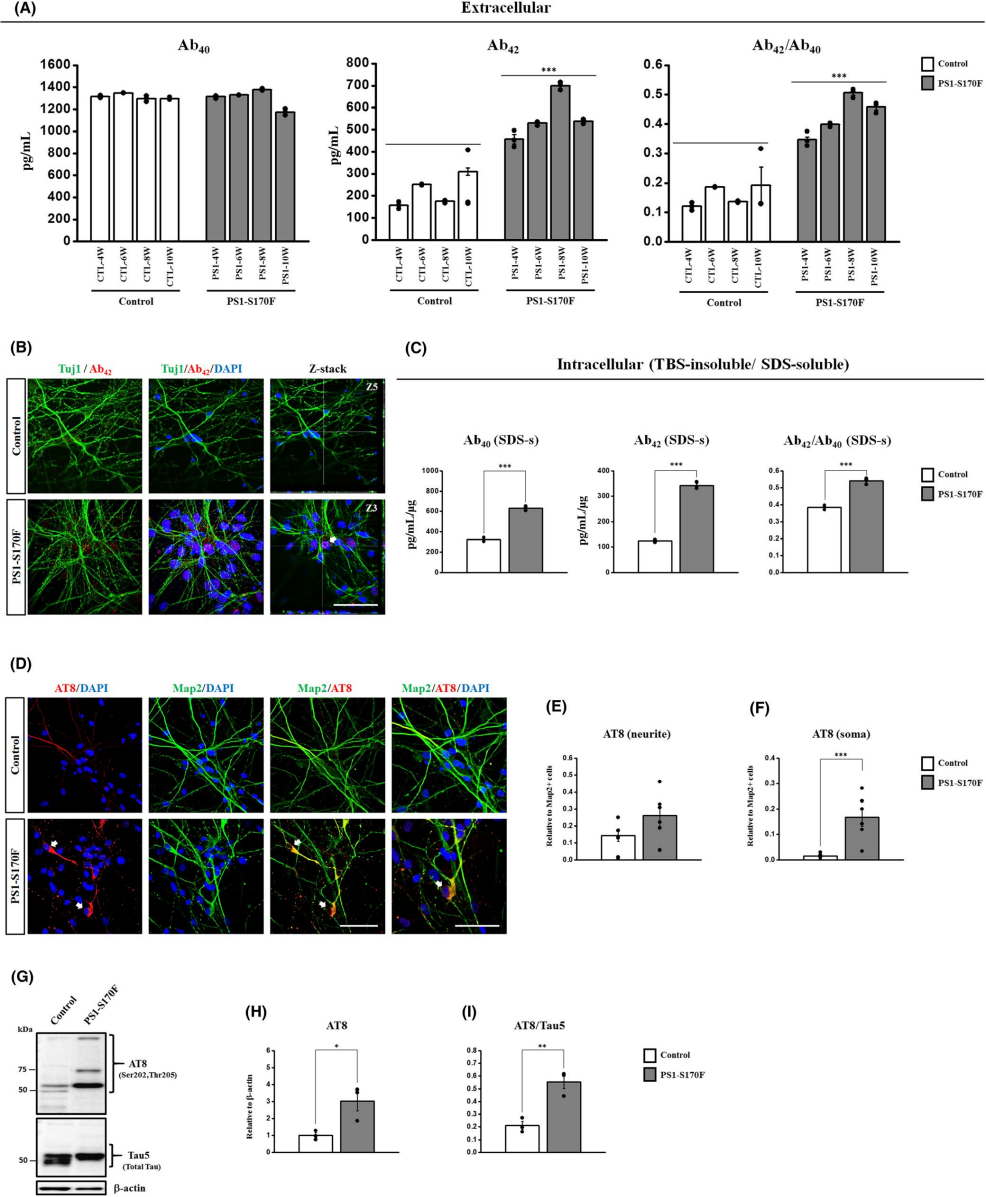
Elevated Aβ and *p*‐Tau levels in the PS1‐S170F iPSC‐derived neurons. A, ELISA detection of Aβ_40_ and Aβ_42_ secreted from the control and PS1‐S170F iPSC‐derived neurons into the medium (extracellular), B, Immunocytochemical analysis showing the expression of Aβ deposits using an antibody against Aβ_42_ (red), co‐stained with Tuj1 (green) and DAPI (blue) at 10 wk of neuronal differentiation. The bottom panels of right side show the z‐stack images of the Aβ_42_‐positive Aβ deposits (indicated as arrow) in PS1‐S170F iPSC‐derived neurons. Scale bar: 10 µm. C, TBS‐insoluble/SDS‐soluble intracellular Aβ_42_ and Aβ_40_ levels were measured in a total of 1 µg proteins using ELISA from 10‐wk‐differentiated neurons. D, Immunocytochemical analysis showing the expression of AT8 (*p*‐Tau) (red) and MAP2 (green), counter‐stained with DAPI (blue) in the iPSC‐derived neurons at 10 wk of neuronal differentiation. Expression of AT8 (*p*‐Tau) was indicated in the soma (arrow) and the neurites (arrowhead). Scale bar: 10 µm. E, F, Quantification of the immunocytochemical analysis, normalized against MAP2‐positive cells. G‐I, Western blot analysis showing an increase of AT8 and the ratio of AT8/ Tau5 in the PS1‐S170F iPSC‐derived neurons. *p* <.05 (*), *p* <.01 (**) and *p* <.001 (***)

To investigate the Aβ levels in the control and PS1‐S170F iPSC‐derived cortical neurons, we measured Aβ_40_ and Aβ_42_ levels secreted from iPSC‐derived neurons into the medium (extracellular) at 48 hours after the last medium change from 4 to 10 weeks of neuronal differentiation. No difference was noted in Aβ_40_ levels. However, PS1‐S170F iPSC‐derived neurons exhibited a dramatic increase in Aβ_42_ levels (over 2‐fold) from 4 weeks after differentiation. Importantly, the ratio of Aβ_42_/Aβ_40_ was significantly increased (over 2‐fold) in the PS1‐S170F iPSC‐derived neurons, compared with the control (Figure 2A).

To detect the Aβ deposits, the iPSC‐derived neurons were stained with anti‐Aβ_42_ antibody at 10 weeks of neuronal differentiation. Notably, confocal microscopy images showed a robust increase in extracellular Aβ_42_ deposits in the PS1‐S170F iPSC‐derived neurons, compared with the control (Figure 2B).

To measure the insoluble Aβ, we extracted TBS‐insoluble/SDS‐soluble fractions described previously.^32^, ^33^ TBS‐insoluble/SDS‐soluble intracellular Aβ_42_ and Aβ_40_ levels were measured in a total of 1µg proteins using ELISA from 10 weeks of neuronal differentiated cortical neurons. Levels of the intracellular Aβ_42_ and the ratio of Aβ_42_/Aβ_40_ showed a significant increase in the PS1‐S170F iPSC‐derived neurons. Surprisingly, we also observed a marked increase of TBS‐insoluble Aβ_40_ levels in the PS1‐S170F iPSC‐derived neurons, compared with the control (Figure 2C).

Taken together, we demonstrated that the PS1‐S170F patient iPSC‐derived neurons exhibited significant increases in extracellular Aβ levels from 4 weeks of neuronal differentiation, as well as TBS‐insoluble/SDS‐soluble intracellular Aβ levels at 10 weeks of neuronal differentiation.

### 3.4 Elevated phospho‐tau levels in the PS1‐S170F iPSC‐derived neurons

In mature neurons, tau protein normally exists as a soluble form in axons. However, in AD the tau protein is hyperphosphorylated and phosphorylated tau (*p*‐Tau) protein is abnormally accumulated in dendrites and cell bodies.^34^, ^35^ To detect the *p*‐Tau levels in the iPSC‐derived neurons, we performed immunocytochemical analysis using antibodies against AT8 (phosphorylated at Ser202/Thr205) and found that PS1‐S170F iPSC‐derived neurons exhibited significant increase of AT8 expression in neurites and soma, compared with the control (Figure 2D‐F). Subsequently, we performed Western blot analysis to measure the AT8 and Tau5 (total tau) expression. We detected the typical bands of AT8 in the fractions from the PS1S170F iPSC‐derived neurons, and the AT8 levels (>50 kDa) were strongly increased in the PS1‐S170F iPSC‐derived neurons compare with the control (Figure 2G‐I).

These results demonstrated that there are high levels of hyperphosphorylated tau which localizes to the cell bodies in the PS1‐S170F iPSC‐derived neurons compared with the control at 10 weeks of neuronal differentiation.

### 3.5 Impaired axonal transport of mitochondria and the balance of mitochondrial fusion and fission in the PS1‐S170F iPSC‐derived neurons

**FIGURE 3:**
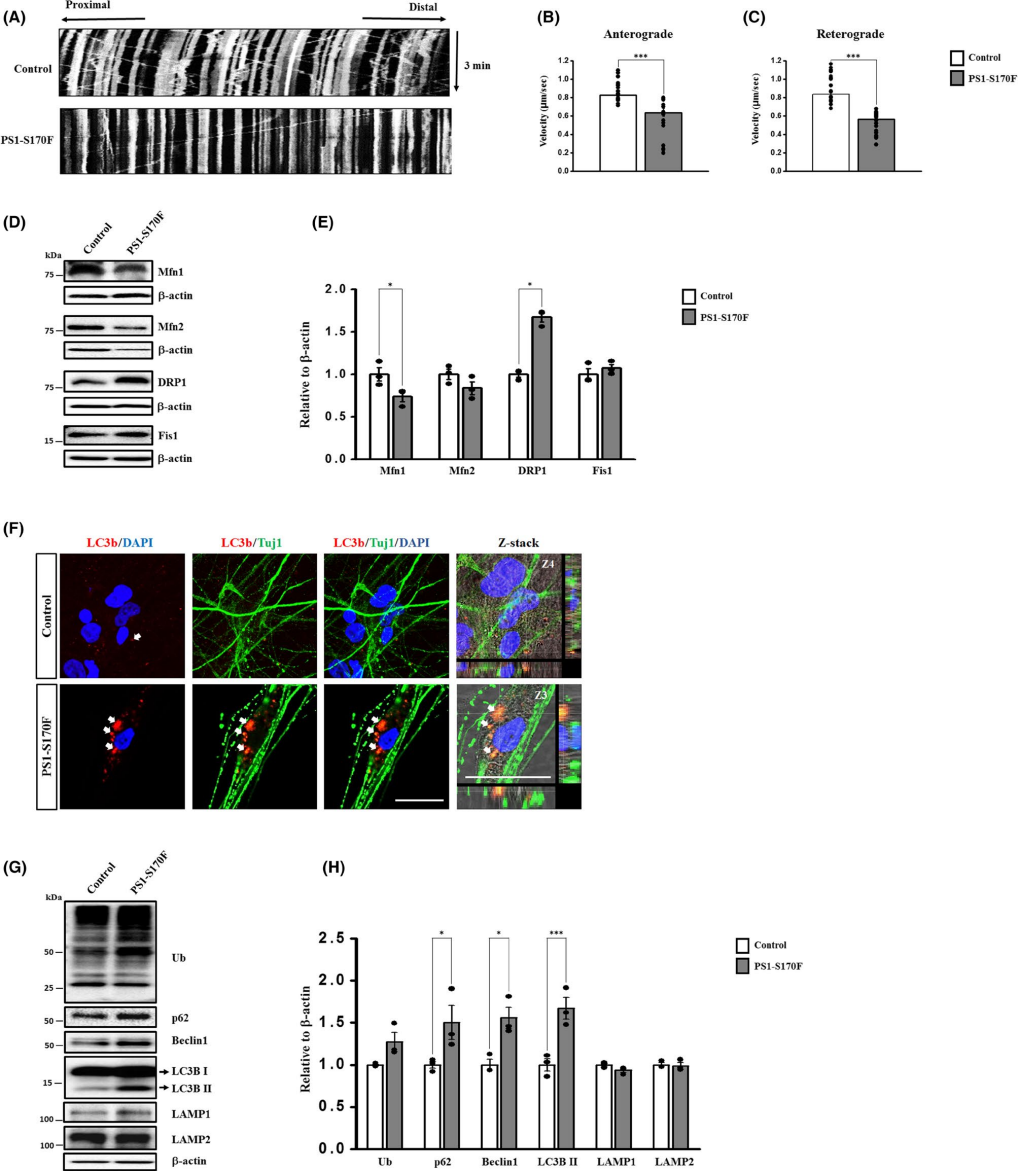
Impaired mitochondrial dynamics and autophagy in PS1‐S170F iPSC‐derived neurons. A, Representative kymographs for mitochondrial movements. Cells were live stained using Mito‐tracker at 10 wk of neuronal differentiation. Average of (B) anterograde and (C) retrograde velocity were analysed separately in motile mitochondria from iPSC‐derived neurons. In both cases, significant decrease was observed in the PS1‐S170F iPSC‐derived neurons compared with the control. D, Representative Western blot images from three independent experiments. E, Quantification of marker proteins associated with mitochondrial fusion (Mfn1 and Mfn2) and fission (DRP1 and Fis1). Note that fusion‐related protein Mfn1 was decreased, whereas fission‐related protein DRP1 was increased in PS1‐S170F iPSC‐derived neurons compared with the control. F, Immunocytochemical staining showing the expression of LC3b (red), Tuj1 (green) and DAPI (blue) in control and AD iPSC‐derived neurons at 10 wk of neuronal differentiation. Note that AD patient iPSC‐derived neurons exhibit a dramatic increase of LC3b in the periphery of soma area (arrow), compared with the control group. Scale bar: 10 µm. G, Representative Western blot images from three independent experiments. H, Quantification of the expression of ubiquitination (Ub) and autophagy‐related proteins (p62, Beclin1, LC3B, LAMP1 and LAMP2). Note that levels of p62, Beclin1 and LC3b were increased in PS1‐S170F iPSC‐derived neurons. *p* <.05 (*) and *p* <.001 (***)

Recent studies reported that high levels of Aβ and *p*‐Tau were found to impair axonal transport of mitochondria.^36^ To investigate the mitochondrial dynamics, we performed the live cell imaging using Mito‐tracker (Ds‐Red) at 10 weeks of neuronal differentiation. We found that the PS1‐S170F iPSC‐derived neurons showed a reduced mitochondrial movement, compared with that of the control (Movies S1 and S2). Kymograph analysis on mitochondria was performed using KymographClear, and the quantification analysis of mitochondria velocity was conducted using KymographDirect (Figure 3A). Both the anterograde and retrograde velocities exhibited a significant decrease in the PS1‐S170F iPSC‐derived neurons, compared with the control (Figure 3B,C). The mitochondrial dynamics is determined by the constant process of fusion and fission, and this process is tightly regulated by the balance of fusion and fission‐related proteins.^18^, ^37^ Therefore, we investigated the expression patterns of mitochondrial fusion and fission‐related proteins, including mitochondria fusion‐related protein Mfn1 (membrane proteins mitofusin 1), Mfn2 (membrane proteins mitofusin 2), mitochondria fission‐related proteins DRP1 (dynamin‐related protein 1) and Fis1 (mitochondrial fission 1 protein). Western blot analysis revealed that Mfn1 expression was dramatically reduced in the PS1‐S170F iPSC‐derived neurons. On the other hand, DRP1 expression levels were significantly increased in the PS1‐S170F iPSC‐derived neurons, compared with that of the control (Figure 3D,E). These results strongly suggest that high levels of Aβ and *p*‐Tau may disrupt the mitochondrial transport, through impaired balance of mitochondrial fusion and fission in the PS1‐S170F iPSC‐derived neurons.

### 3.6 Defective autophagy‐related clearance in the PS1‐S170F iPSC‐derived neurons

Autophagy is essential for neuronal survival, as it has an important role in degradative pathway for proteins and organelles. In AD, defective autophagy is evident as the autophagy vacuoles (AVs) are accumulated in the brain tissues of AD patients, which are correlated with the presence of neuritic plaques and filamentous tau.^22^, ^38^ Also, it has been reported that increased autophagy induction and defective clearance of Aβ generating AVs results in Aβ accumulation.^20^, ^21^, ^39^ Therefore, to investigate whether the autophagosome is also abnormally accumulated in the PS1‐S170F iPSC‐derived neurons, we performed immunocytochemical analysis at 10 weeks of neuronal differentiation which showed very high levels of Aβ_42_ production and *p*‐Tau. We found that the PS1‐S170F iPSC‐derived neurons exhibited a dramatic increase of LC3b in the periphery of soma area, compared with the control (Figure 3F). In addition, we measured expression levels of autophagy‐related proteins, including Ub (Ubiquitin), p62 (SQSTM1, sequestosome 1; cargo protein marker), Beclin1 (autophagosome membrane formation‐related protein), LC3b (light chain 3; autophagosome formation marker), LAMP1 (lysosome‐associated membrane protein1) and LAMP2 at 10 weeks of neuronal differentiation. Western blot and its quantification analysis revealed that p62, Beclin1 and the canonical autophagosome marker LC3b were significantly increased in the PS1‐S170F iPSC‐derived neurons (Figure 3G,H), suggesting that LC3b‐dependent autophagy was activated at 10 weeks of neuronal differentiation. However, there was no change in the lysosomal markers, including LAMP1 and LAMP2 (Figure 3G,H). Taken together, even if the LC3b‐based autophagy system was activated, PS1‐S170F iPSC‐derived neurons showed very high levels of Aβ_42_ and *p*‐Tau expression, implying that the PS1‐S170F iPSC‐derived neurons are impaired in autophagy‐related clearance system at 10 weeks of neuronal differentiation.

### 3.7 Reduction of Aβ and *p*‐Tau levels by disease‐modifying drug candidate (BACE1 inhibitor)

**FIGURE 4:**
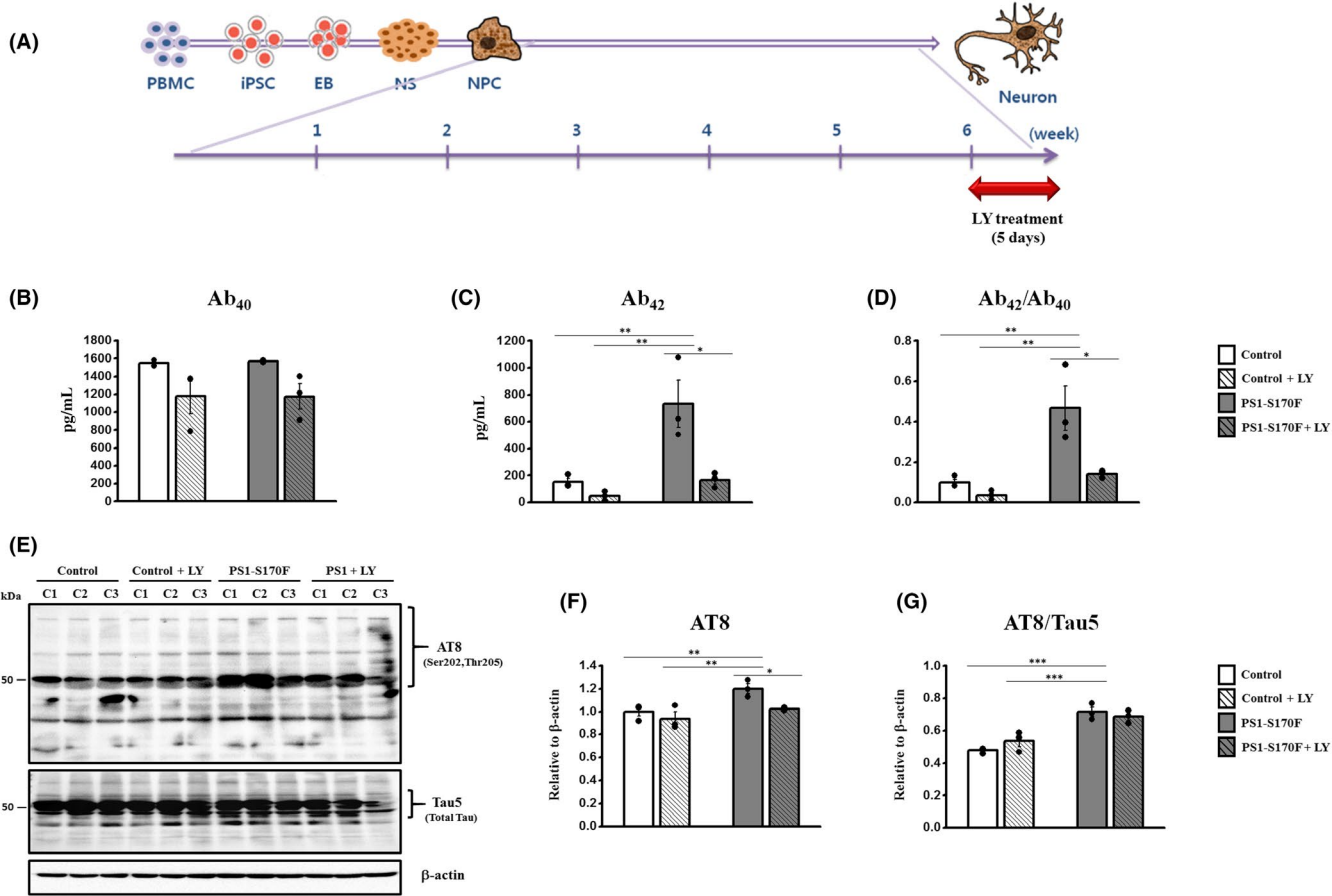
Reduction of Aβ and *p*‐Tau levels by candidate drug treatment. A, Schematic representation of drug treatment for iPSC‐derived neurons. The drug was treated for 5 d after 6 wk of neuronal differentiation. Levels of (B) Aβ_40,_ (C) Aβ_42_ and (D) the ratio of Aβ_42_/Aβ_40_ were measured after LY2884721 (BACE1 inhibitor) treatment. Levels of Aβ_42_ and the ratio of Aβ_42_/ Aβ_40_ showing significant decrease in iPSC‐derived neurons after LY2884721 treatment. E, Representative immunoblot against *p*‐Tau (AT8), and (F, G) quantification of the immunoblot against *p*‐Tau (AT8) showing dramatic decreases in PS1‐S170F iPSC‐derived neurons after LY2884721 treatment. *p* <.05 (*), *p* <.01 (**) and *p* <.001 (***)

Aβ is produced by the sequential cleavage of APP by β‐site APP cleaving enzyme 1 (BACE1) and γ‐secretase. Therefore, these two enzymes are regarded as the most important targets for candidate drugs of AD, and previous studies have shown the rescue effects on Aβ production when the familial AD patient iPSC‐derived neurons were treated with BSI (BACE1 inhibitor).^12^, ^40^ For candidate drug treatment, we treated the PS1‐S170F iPSC‐derived neurons with 5 µmol/L LY‐2886721, a BACE1 inhibitor, for 5 days after 6 weeks of neuronal differentiation and investigated the effects on Aβ and *p*‐Tau levels (Figure 4A). Importantly, the levels of Aβ_42_ and the ratio of Aβ_42_/Aβ_40_ were shown to be decreased significantly after LY2884721 treatment (Figure 4B‐D). Furthermore, the expression of *p*‐Tau (AT8) was also shown to be decreased dramatically in the PS1‐S170F iPSC‐derived neurons after LY2884721 treatment (Figure 4E‐G). These results demonstrate that our PS1‐S170F iPSC‐derived neurons are suitable for AD drug screening, and as such, they can serve as a useful resource in the future.

## 4 DISCUSSION

In an attempt to understand AD pathogenesis, we have established and characterized the patient iPSC line from an AD patient carrying PS1‐S170F mutation. PS1‐S170F mutation has been identified in several independent families with very early‐onset dementia in the late 20s.^3^, ^4^, ^5^ An autopsy study from three AD patients carrying PS1 S170F mutation showed extensive neuronal loss, abundant neuritic plaques and neurofibrillary tangles involving the entire neocortex.^5^ Another autopsy study from a 28‐year‐old AD patient with PS1 S170F mutation also showed severe Aβ deposition with neurofibrillary tangles in the cerebral cortex.^4^ Although we could not obtain brain tissue from our patient, we could assume AD pathology in the patient's brain as he showed PS1‐S170F mutation, rapid cognitive decline, family history of early‐onset dementia and abnormal neuroimaging findings (cortical atrophy especially in the bilateral parietal areas and hypometabolism in the bilateral temporo‐parietal areas). Although PS1‐S170F is a well‐known mutation causing familial AD, iPSC generation and characterization results have not been reported previously.

We found that the differentiated neurons exhibited the spontaneous repetitive AP of currents clamp mode at 10‐12 weeks after differentiation in all of the iPSC‐derived neurons (Figure S2D‐H). These results strongly suggest that the differentiated neurons generated the neuronal signal responses and were matured into functional neurons in vitro at the 10 weeks of neuronal differentiation. We also found that the PS1‐S170F iPSC‐derived neurons showed a significant increase of extracellular and TBS‐insoluble/SDS‐soluble intracellular Aβ_42_ levels from 4 weeks of neuronal differentiation and exhibited very high levels of Aβ_42_ and the ratio of Aβ_42_/Aβ40 at 10 weeks of neuronal differentiation (Figure 2A‐C). Previous studies demonstrated that brain tissues from severe Braak stages of AD patients^37^, ^41^ and neural cells with PSEN1 and APP mutation showed more multiple bands in *p*‐Tau (>50 kDa), compared with the controls.^32^ We also detected the abnormally accumulated *p*‐Tau (AT8) in the cell bodies and multi‐bands of AT8 in the fractions (>50 kDa) that may indicate the hyperphosphorylated‐tau^32^, ^37^, ^41^ in the PS1‐S170F iPSC‐derived neurons. In addition, the AT8 levels (>50 kDa), which are maybe related to the localization in the cell bodies were strongly increased in the PS1‐S170F iPSC‐derived neurons, compared with the control (Figure 2D‐I) at 10 weeks of neuronal differentiation. These results support the hypothesis that accumulation of Aβ peptides may result in the aggregation of hyperphosphorylated‐tau proteins in the cell bodies.^27^, ^28^, ^32^

Mitochondrial dysfunction is a prominent feature of AD. Mitochondria are a highly dynamic organelle, and the intracellular population at any time point is derived from a balance between fission and fusion.^17^, ^36^ We observed dramatic decrease in movement of anterograde and retrograde mitochondrial velocity in the PS1‐S170F iPSC‐derived neurons (Figure 3A‐C and Movies S1 and S2. Western blot analysis revealed that fission associated markers were increased, whereas fusion‐related markers were markedly decreased in PS1‐S170F iPSC‐derived neurons compared with the control (Figure 3D,E). As extracellular Aβ and phosphorylated tau are implicated in mitochondrial fission and fusion in humans, we speculated that the increased secretion and aggregation of Aβ and phosphorylated forms of tau protein caused this defective mitochondrial axonal transport and imbalance of mitochondrial fission and fusion. Many reports suggest the possibility of defective autophagy in AD. It has been demonstrated that autophagy vacuoles (AVs) accumulate in human AD brains and are related to the presence of neuritic plaques and filamentous tau.^22^, ^38^ In this study, we observed significantly increased active forms of LC3b‐II in the PS1‐S170F iPSC‐derived neurons at 10 weeks of neuronal differentiation (Figure 3F‐H), which strongly suggests that high levels of Aβ and *p*‐Tau may impair the autophagy‐related clearance system at 10 weeks of neuronal differentiation.

BACE1 and γ‐secretase are the most important targets for candidate drugs of AD and previous studies showed the rescue effects on Aβ production treated with BSI (BACE1 inhibitor) in familial AD patient iPSC‐derived neurons.^12^, ^40^ However, strong inhibition of γ‐secretase has caused side effects after long‐term treatment.^42^, ^43^ BACE1 was considered as a more preferable target for anti‐Aβ drugs, and several pharmaceutical companies are currently undergoing clinical trials for BACE1 inhibitors (BSIs).^12^, ^40^ As AD is heterogenous and drug response may vary in each individual, we need a reliable screening system using patient‐specific human cells for investigating the therapeutic effects of the candidate drugs on each individual. Therefore, after we confirmed that PS1‐S170F iPSC‐derived neuron exhibited the typical AD pathological features, we performed a proof‐of‐concept experiment to demonstrate the suitability of our AD patient‐derived iPSC lines for candidate drug screening. In this study, we selected LY‐2886721, a BACE1 inhibitor, to study its effects on Aβ_42_ and *p*‐Tau levels. First of all, levels of Aβ_42_ and the ratio of Aβ_42_/Aβ_40_ showed a significant decrease after 5 µmol/L of LY2884721 treatment, compared with vehicle (VC) neurons (Figure 4B‐D). Interestingly, *p*‐Tau (AT8) also showed a dramatic decrease in the PS1‐S170F iPSC‐derived neurons after LY2884721 treatment (Figure 4E‐G). However, there was no significant rescue effect on mitochondria and autophagy dysfunction after LY2884721 treatment (data not shown). Taken together, these results strongly suggest that, although clinical trials using BACE1 inhibitor have not succeeded yet, the ADAD patients with PS1‐S170F might benefit from this drug treatment.

In summary, we generated an induced iPSC line from AD patient carrying PS1‐S170F mutation and then differentiated them into functional cortical neurons. We found high levels of extracellular/intracellular Aβ and *p*‐Tau in the PS1‐S170F iPSC‐derived neurons compare with the control. We next investigated impaired mitochondrial dynamics and balance of fusion and fission in PS1‐S170F iPSC‐derived neurons. Furthermore, we also demonstrated defects of autophagy‐related clearance PS1‐S170F iPSC‐derived neurons. In addition, levels of Aβ and *p*‐Tau exhibited dramatic reduction by LY‐2886721 (BACE1 inhibitor) treatment in the PS1‐S170F iPSC‐derived neurons. Taken together, we have characterized the pathological features of AD patient carrying mutation for PS1‐S170F using iPSC technology and observed a good response to a candidate drug. Our study is worthwhile as we demonstrated that iPSC‐derived neuron may serve as a disease model and drug screening which may pave way for personalized therapy for AD patients in the future.

## CONFLICT OF INTEREST

JS is the founder and CEO of iPS Bio, Inc.

## AUTHOR CONTRIBUTIONS

LL, HJK, DLN and JS were responsible for the study concept and design. LL, HJK JHR, MK, WK, YK, HH, JC, MN, TY, CPH and SWS were responsible for data acquisition. LL, HJK and JS performed data analysis and manuscript writing. JS and DLN finalized the manuscript. LL and HJK contributed equally.

## ETHICS APPROVAL AND CONSENT TO PARTICIPATE

Each institutional review board of CHA University, Samsung Medical Center, and Asan Medical Center approved the study protocol [1044308‐201612‐BR‐031‐05], and the informed consents were obtained from participants.

## Supporting information

Fig S1‐S2

Movie S1

Movie S2

## Data Availability

The data that support the findings of this study are available from the corresponding author upon reasonable request.
