# Supplementary material for: Pathological manifestation of the induced pluripotent stem cell‐derived cortical neurons from an early‐onset Alzheimer's disease patient carrying a presenilin‐1 mutation (S170F)
Source: Cell Prolif. 2020 Mar 25;53(4):e12798. doi: 10.1111/cpr.12798 (PMC7162796; doi:10.1111/cpr.12798)
Supplement: Supplementary file 1 — Fig S1‐S2 [file CPR-53-e12798-s001.docx]

**Supporting information file**

**Cell Proliferation**

**Pathological manifestation of the iPSC-derived cortical neurons from an early-onset Alzheimer’s disease patient carrying a presenilin-1 mutation (S170F)**

Ling Li, Hee Jin Kim, Jee Hoon Roh, Minchul Kim, Wonyoung Koh, Younghoon Kim, Hyohoon Heo, Jaehoon Chang, Mahito Nakanishi, Taeyoung Yoon, Chang Pyo Hong, Sang Won Seo, Duk Lyul Na, Jihwan Song.

**
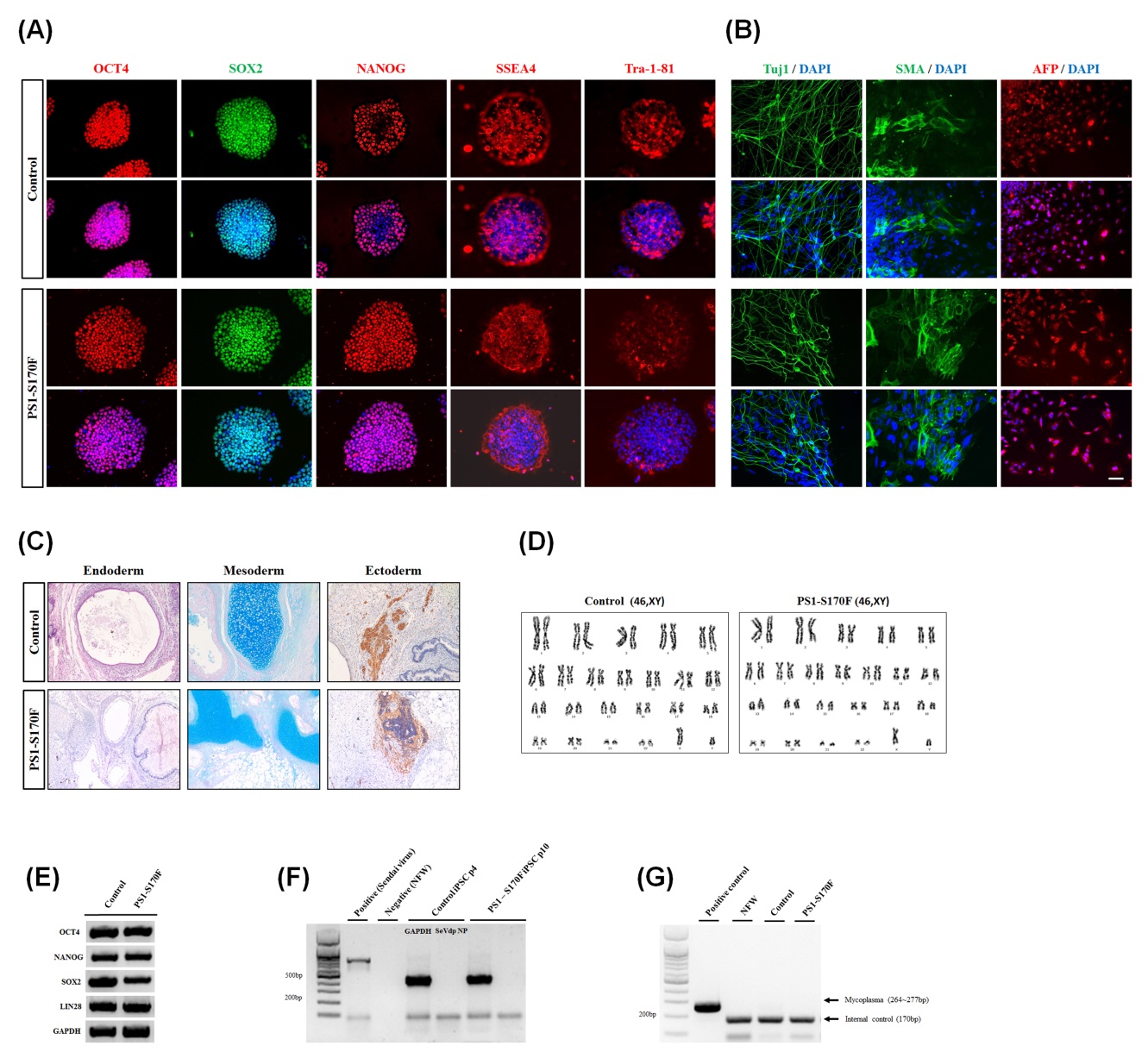
**

**Figure S1. Characterization of control and AD (PS1-S170F) patient-derived iPSC line**

**(A)** Established iPSCs from control and AD patients showing the expression of pluripotent stem cell markers, such as OCT4 (red), SOX2 (green), SSEA4 (red) and TRA-1-81 (red). **(B)** Immunocytochemistry showing the potential of iPSC line to form three germ layers, including ectoderm (TUJ1, green), mesoderm (SMA, green), and endoderm (AFP, red). Scale bar: 100µm. **(C)** *In vivo* (teratoma) formation with all three-germ layers: gut-like epithelium (endoderm), cartilage (mesoderm) and Tuj1-positive (ectoderm). **(D)** Karyotype analysis of the control and PS1-S170F iPSC lines. **(E)** Reverse-transcription PCR analysis showing the mRNA expression of pluripotent markers in control and PS1-S170F iPSC lines. **(F)** Reverse-transcription PCR analysis showing the absence of integration of the Sendai virus vectors. **(G)** PCR analysis showing no contamination by mycoplasma.

**
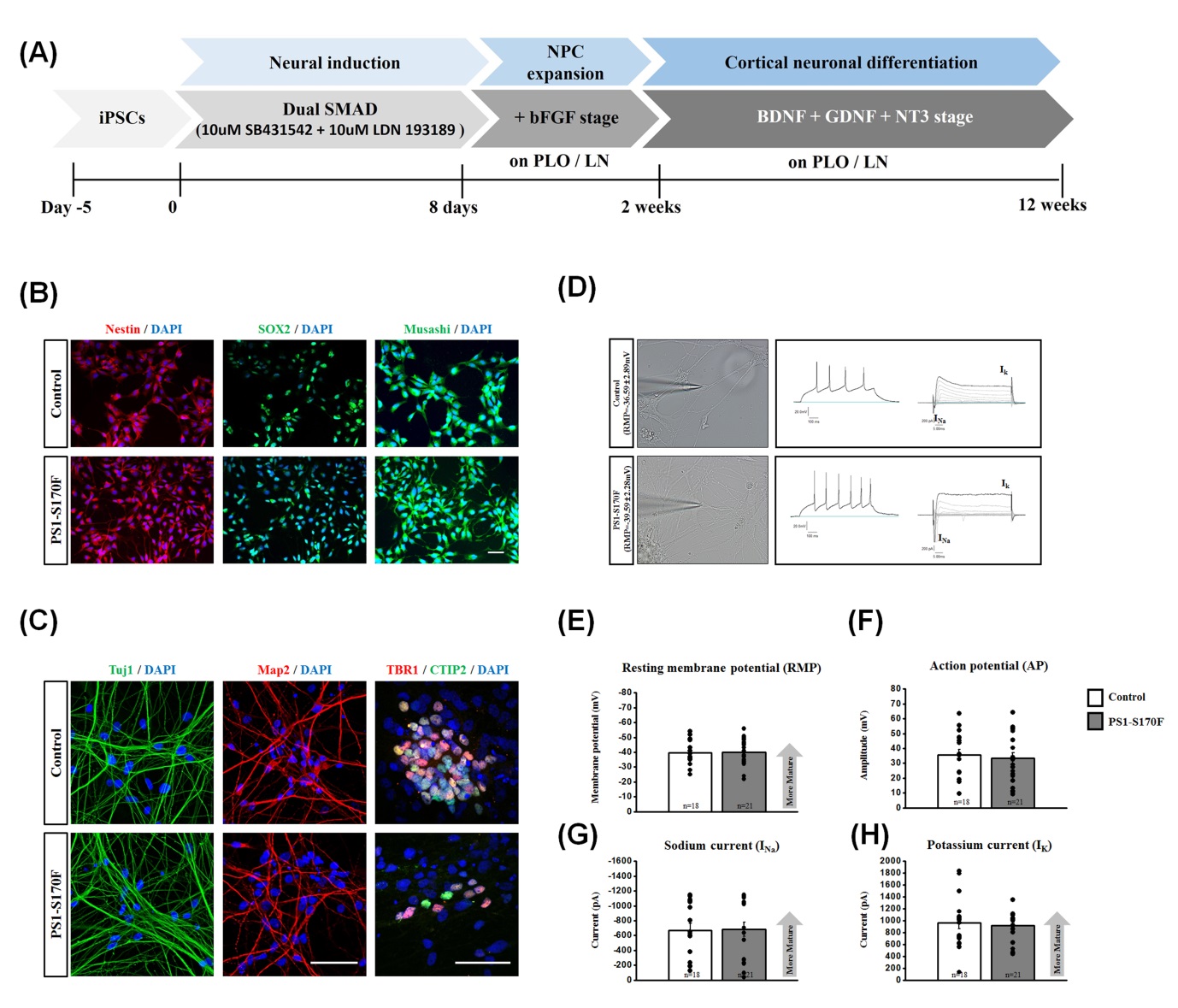
**

**Figure S2. Cortical neuron differentiation and electrophysiological analysis in AD iPSC-derived neurons**

**(A)** Schematic diagram showing our cortical neuron differentiation protocol. **(B)** Immunofluorescence analysis of control and PS1-E120K iPSC-derived NPCs, showing the expression of NPC markers, such as Nestin (green), SOX2 (red) and Musashi (green) with DAPI (blue). **(C)** Immunofluorescence analysis of control and AD-iPSC-derived cortical neurons, labelled for neurons (Tuj1 [red], Map2 [red]) and more specifically cortical neurons (TBR1 [red] and CTIP2 [green]) at 10 weeks after differentiation. Scale bar: 50µm. **(D)** DIC photograph of differentiated neurons and the traces obtained from iPSC-derived neurons during current clamp (CC) and voltage clamp (VC). Peak of sodium (I_Na_) and potassium (I_K_) current were across all cell lines with each cell lines. Quantification of **(E)** resting membrane potential (RMP), **(F)** amplitude of AP, **(G)** I_Na_ current and **(H)** I_K_ current showing no obvious differences in control and AD patient iPSC-derived neurons.
